# Retraction: Assessment of expression of regulatory T cell differentiation genes in autism spectrum disorder

**DOI:** 10.3389/fnmol.2025.1586805

**Published:** 2025-03-17

**Authors:** Frontiers Editorial Office

The journal retracts the July 4^th^ 2022 article cited above.

Following publication, concerns regarding potential undisclosed conflicts of interest were raised by a reader. An investigation was conducted in accordance with Frontiers' policies, after which Frontiers found substantial evidence of undisclosed conflicts of interest that undermined the integrity of the peer review process. Additionally, following the Chief Editor's assessment, it was determined that the validity of the content was significantly compromised by the inclusion of retracted references. As the scientific integrity of the article cannot be guaranteed, and in adherence to the recommendations of the Committee on Publication Ethics (COPE), the article is retracted.

This retraction was approved by the Chief Editors of Frontiers in *Molecular Neuroscience*. The authors do not agree to this retraction.

Frontiers would like to thank Alexander Magazinov for contacting the journal regarding the published article.

